# The Effectiveness of Aloe Vera on Relief of Irritation and Nipple Pain in Lactating Women: Systematic Review and Meta-Analysis

**DOI:** 10.1155/2022/7430581

**Published:** 2022-11-07

**Authors:** Azam Maleki, Samaneh Youseflu

**Affiliations:** ^1^Maternal and Child Health, Social Determinants of Health Research Center, Zanjan University of Medical Sciences, Zanjan, Iran; ^2^Reproductive Health, Department of Midwifery and Reproductive Health, Isfahan University of Medical Sciences, Isfahan, Iran

## Abstract

**Background:**

*Aloe vera* is one of the herbal products with anti-inflammatory, antioxidant, moisturizing, bactericidal, anti-viral, and anti-fungal effects that were used to relieve pain and irritation. The aim of the current systematic review and meta-analysis is to determine the effect of *Aloe vera* on the relief of irritation and nipple pain in lactating women.

**Methods:**

A search was carried out in four English electronic databases including Scopus, Embase, PubMed, and Web of Science until November 2021. All clinical trials that assessed the effect of *Aloe vera* on the relief of irritation and nipple pain in lactating women were included. The study's risk of bias was assessed using the Cochrane risk of bias checklist. Study heterogeneity was determined using the *I*^2^ statistic and publication bias using Begg's and Egger's tests. Results of the random-effects meta-analysis were presented using standard mean difference (SMD) with 95% confidence intervals (CIs). Data were analyzed using STATA software version 16 MP.

**Results:**

In total, 7 articles with 1670 subjects were included in the meta-analysis. Overall, we found a positive impact of *Aloe vera* on reducing breast pain (pooled SMD= −0.45; CI= −0.83, −0.07, *P* value <0.02) and irritation (pooled SMD= −0.48; CI= −0.64, −0.32, *P* value<0.001) in lactating women. There is a high heterogeneity among pain studies (*I*^2^= 86%) but was low within irritation studies (*I*^2^= 26%).

**Conclusion:**

Our result showed that *Aloe vera* can be considered a choice for relieving breast pain or irritation in lactating women compared with routine care or another treatment. Considering the limited number of studies conducted on this topic and the low sample size, future studies with a larger sample will be required to draw better conclusions.

## 1. Introduction

Breastfeeding is considered the most important factor in reducing the infant mortality rate, as well as has many benefits for maternal health. Accordingly, the World Health Organization and UNICEF recommend that every infant should have exclusive breastfeeding for at least six months after birth and it should continue for 2 years [1, 2]. Despite their recommendation, only 37% of infants aged less than six months are exclusively breastfed in low-income and middle-income countries [3].

Treating breastfeeding is an art that requires many skills and knowledge, especially for new mothers. Improper attachment of the infant to the breast, along with the growth factor of mucus present in infant saliva and breast milk, can be associated with nipple damage [4, 5]. Nipple pain is a protective response of damaged tissue following vasospasm of the nipple tissue, reduction of capillary blood supply, swelling, and oedema [6]. Nipple pain, with a prevalence of 34 and 97%, is one of the most common complaints among breastfeeding mothers and the second reason for premature discontinuation of breastfeeding after feeling insufficient milk secretion [7, 8]. Breast and nipple pain can reduce oxytocin and milk secretion by increasing catecholamine secretion, as well as increasing cytokines and proinflammatory factors [9].

Various methods have been proposed for the prevention and treatment of nipple fissures, such as correcting breastfeeding techniques, rubbing milk on the nipple, glycerin pads, using topical and collagenase ointments, hydrogel, honey, and herbal medicine, neither of which is superior to the other [5].

The natural products in the diet play an important role in the prevention and treatment of many chronic conditions such as diabetes, cancer, cardiovascular disease, and hypertension [8, 10, 11]. Some studies support the importance of food-derived natural compounds such as flavonoids, terpenes, alkaloids, phenols, carotenoids, palmitoylethanolamide, zerumbone, dehydrocorybulbine, *β*-caryophyllene, berberine, chlorogenic acid, and isoflavones in the relief of neuropathic pain through multiple mechanisms such as regulating the estrogen receptor, antioxidant, and anti-inflammatory activities [10, 12].

Today, herbal medicine is widely used in the fields of obstetrics and gynaecology. *Aloe vera* is one of the herbal products with anti-inflammatory, antioxidant, moisturizing, bactericidal, anti-viral, and anti-fungal effects that was used to relieve pain and irritation [2, 13, 14]. There are high amounts of molecular material, water, polysaccharides, sugars, minerals, proteins, lipids, phenolic compounds, as well as many vitamins such as *A*, *C*, *E*, *B*1, *B*2, and *B*9 in *Aloe vera* [15]. In addition, there are many enzymes such as bradykinase, carboxypeptidase, cellulase, amylase, catalase, and oxidase in this plant. These enzymes by blocking the synthesis of bradykinin, cyclooxygenase-2, and thromboxane synthase can reduce the pain intensity [16]. Carboxypeptidase inactivates bradykinase at the wound site and exerts anti-inflammatory and pain-relieving effects. The anti-inflammatory effects of this plant are attributed to the presence of salicylic acid (effective in inhibiting the formation of bradykinin and histamine) and the arachidonic acid oxidation pathway through cyclooxygenase [17]. Magnesium lactate in the *Aloe vera* plant prevents the histamine reaction, therefore relieving itching and skin irritation [18]. The previous study reveals that anthraquinones, allantoin, and other polysaccharide compounds in *Aloe vera* inhibit histamine and bradykinin synthesis, thereby reducing wound pain severity [19]. Also, the phenolic compound of aloe-vera can be reduced edema by a reduction in the level of prostaglandins [20]. Moreover, *Aloe vera* increases the number of fibroblasts and collagen, which can accelerate wound healing [21].

Many studies show a positive impact of *Aloe vera* on the relief of irritation and pain in the nipple, but there is a controversy in their results. Therefore, this study as a first systematic review and meta-analysis was conducted to determine the effect of *Aloe vera* on the relief of irritation and nipple pain in lactating women.

## 2. Methods

### 2.1. Search Strategy, Data Sources, and Data Extraction

This systematic review and meta-analysis were conducted based on the Preferred Reporting Items for Systematic reviews and Meta-Analyses (PRISMA) statement. A comprehensive and systematic search was performed in English and Persian electronic databases including Medline, PubMed, Web of Science, Scopus, Embase, Cochrane Database of Systematic Reviews (CDSR), CINAHL, Sid, IRANDOC, and Mag-Iran for the period up to November 20, 2020, using the following search strategies following the Mesh browser keywords and free-text words: (“Aloe” [mh] OR “Aloe vera gel” [mh] OR “Aloe” [tiab] OR “Aloe vera gel” [tiab] OR “Herbal Medicine” [mh] OR “herbal medicine” [tiab] OR “herb” [tiab]) AND (“Nipples” [mh] OR “nipple trauma” [tiab] OR “nipple injuries” [tiab] OR “nipple wound” [tiab] OR “nipple fissure” [tiab] OR “ nipple sore” OR “nipple pain” Or “irritation” OR “Nipples” [tiab] OR “breast pain” [tiab] OR “Breast Feeding” [mh] OR “Breast Feeding” [tiab] “Breastfeeding” [tiab] OR “Breast Fed” [tiab] OR “Breastfed” [tiab]). Moreover, the manual approaches, in particular, hand-searching and perusing the bibliographies of retrieved articles were performed to find additional articles. Also, various grey literature databases (such as Google Scholar, Open Grey SIGLE, Global Index Medicus (GIM), NTIS, World Cat, and UW Libraries Search), theses and dissertations, as well as conference proceedings were scanned for collecting unpublished data.

Using Endnote software ver.X9 duplicate, studies in different databases were eliminated by following the PRISMA Systematic Review Flowchart. The titles and abstracts of all articles were scanned. In the next step, we checked the full text of the studies to identify the articles that met the inclusion criteria. Also, to reduce the potential risk of bias, two reviewers performed the literature review, data selection, and quality assessment of data independently. Disagreements of the investigator were resolved through discussion or consultation with the third reviewer to reach a consensus [22].

After finding the full texts, data of each article were extracted using a structured form that included the author's name, publication date, location of study, setting, the sample size of each group, type of intervention in each group, measurement tool, results, and *P* value. The quantitative data were entered into STATA version 15.

### 2.2. Inclusion and Exclusion Criteria

Clinical trial studies using *Aloe vera* that have a control group with placebo or comparison with other treatments in the prevention and treatment of irritation and nipple pain were included in the systematic review.

Studies were excluded if they were at least one of the following criteria: (1) lack of access to full text, (2) unexplained methods, (3) systematic reviews and meta-analysis, animal experiment, cross-sectional, and observational study design, (4) letters, editorials, case reports, review articles, and conference abstracts, and (5) inability to extract the outcome measures.

### 2.3. Outcome

The main outcome of this study was nipple pain and irritation.

#### 2.3.1. Quality Assessment

The risk of bias for each selected study was assessed independently by two investigators (AM and SY) based on seven criteria that are required by Cochrane guidelines for quality assessment of randomized controlled trials [22]. This tool examines the following issues: random sequence generation (selection bias), allocation concealment (selection bias), blinding of the participants and personnel (performance bias), incomplete outcome data (attrition bias), blinding of outcome assessors (detection bias), selective outcome reporting (reporting bias), and other risks of bias. Each item was evaluated using three evaluation options, including low, unclear, and high risk options. Any disagreements in the risk of bias/quality appraisal assessments were discussed by the two authors until a consensus was reached.

#### 2.3.2. Analysis

**Figure 1:**
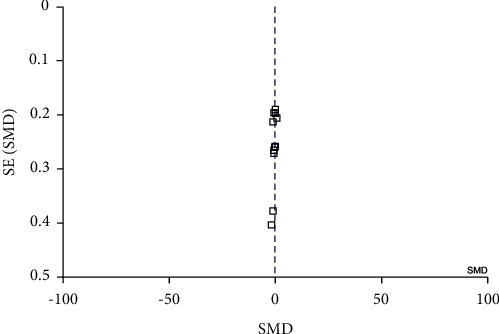
Funnel plot.

STATA software version 16 was used for data analysis. The standardized mean difference or SMD was used to find the effect for quantitative data. The *I*^2^, Tau2, and *χ*^2^ statistics were used to evaluate the heterogeneity of the included studies. To identify the degree of heterogeneity, the following classifications of the *I*^2^ statistic were used: unimportant (0–40%), moderate heterogeneity (30–60%), substantial heterogeneity (50–90%), and high heterogeneity (75–100%) [20]. Due to the high level of heterogeneity, the random effect was applied instead of the fixed effect. Sensitivity analyses were done, when we observe a high level of heterogeneity. Publication bias was evaluated by the funnel plot, as well as Egger's and Begg's tests (Figure 1).

## 3. Results

### 3.1. Description of Included Studies

**Figure 2:**
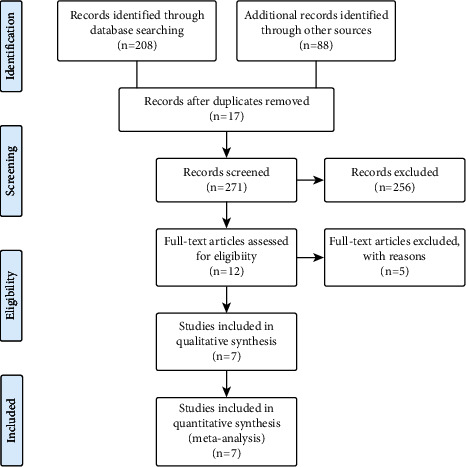
Flow chart of the study selection.

We identified 296 articles by search in the English and Persian major databases including Scopus (105), ISI [5], PubMed (75), Sid [5], Mag-Iran [12], and other sources (88) based on the Mesh browser keywords and free-text words adapted for other databases with no publication year or language limitations. Among the identified articles, we excluded 17 duplicate articles, 271 in the title and abstract review, and finally, one article in the full-text review due to the one-group pretest-posttest design. In total, 7 articles with 1670 subjects were included in the meta-analysis. The selection procedure of the articles is presented in Figure 2. The description of the 7 studies is summarized in Table 1. Of these, 4 studies were conducted in Iran. Two studies were conducted in Indonesia and one study in China. Four articles were published in the English language, one article in the Persian language, and the rest in the Indonesian language. Regarding the type of study, 5 articles were randomized control trials (RCTs) study design, and two articles were in a quasi-experimental design. The source of pain and irritation in four articles was breast soreness, and the rest were milk stasis and breast engorgement in lactating women. The topical gel/extract of *Aloe vera* is applied on breast sores by massage or cold compress. In one study, *Aloe vera* was combined with cactus, and in all studies, the effect of *Aloe vera* was compared with the no treatment/breast milk or lanolin group. The follow-up period for assessing pain and irritation outcomes ranged from immediately after treatment to 14 days after the intervention. In all studies, the pain level was recorded using the Visual Analogue Scale (VAS) for pain and the store scale for breast irritation intensity (Table 1).

### 3.2. Main Result

**Figure 3:**
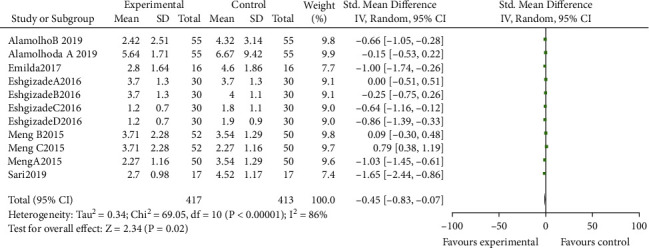
Forest plot of studies that investigated the influence of *Aloe vera* on nipple pain.

**Figure 4:**
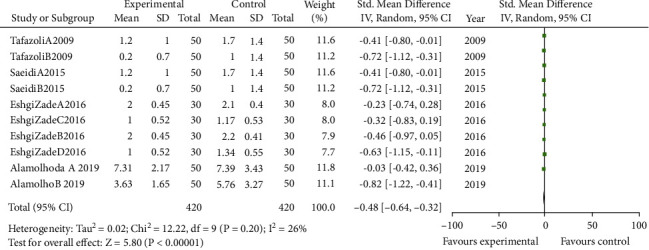
Forest plot of studies that investigated the influence of *Aloe vera* on nipple irritation.

In total, 7 articles with 1670 subjects were included in the meta-analysis [4, 14, 23–27]. The primary outcome was breast pain reported in five articles, and the secondary outcome was breast irritation in lactating women that included four articles. Overall, we found a positive impact of *Aloe vera* on reducing breast pain (pooled SMD = −0.45; CI= −0.83, −0.07, *P* value <0.02) and irritation (pooled SMD= −0.48; CI= −0.64, −0.32, *P* value <0.001) in lactating women. There is a high heterogeneity among pain studies (*I*^2^= 86%) but was low within irritation studies (*I*^2^= 26%) (Figures 3 and 4).

The results reported that no significant publication bias was observed according to Begg's and Eggerʼs tests (*P* = 0.8417and *P* = 0.938), respectively. In Figure 1, the funnel plot showed symmetrical distribution among studies.

### 3.3. Quality of the Included Studies

**Figure 5:**
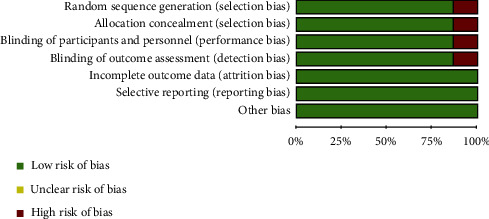
The results of Cochrane Risk of Bias Tool for the evaluation of clinical trial quality.

We used the Cochrane Collaboration recommend tools for assessing the methodological quality of clinical trials. In our study, the quality of the 5 studies was high [4, 14, 25–27] and that of the two studies were low [23, 24] (Figure 5).

### 3.4. Sensitivity Analysis

**Figure 6:**
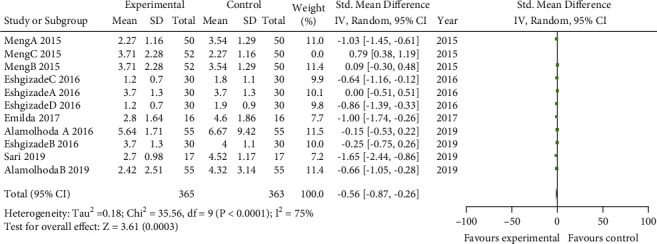
The forest plot of sensitive analysis of nipple pain.

We explored the impact of *Aloe vera* combined with cactus by using a sensitivity analysis and excluding that result. The pool estimates of the sensitive analysis demonstrated that pooled SMD was −0.56; CI= −0.87, −0.26, *P* value<0.001, and *I*^2^= 75% (Figure 6).

## 4. Discussion

To our knowledge, this study is the first meta-analysis to assess the effect of *Aloe vera* on breast pain and irritation in lactating women. Based on the available evidence, the meta-analysis showed that *Aloe vera* can be relieved 0.45-unit pain and 0.48-unit irritation than the no treatment/breast milk lanolin group, and it was statistically significant.

Several systematic reviews were performed regarding the effectiveness of herbal medicine, especially Aloe vera, on wound healing and breast issues (eg., fissures, soreness, and irritation) in the postpartum period. In this regard, in 2017, Niazi et al. conducted a systematic review of 17 clinical trials (2 articles for herbal treatment, 2 articles for drug treatment, and 7 articles for nondrug treatment) to evaluate the effectiveness of various methods (such as silver cup, polyethylene protector, tea bag, phototherapy, laser therapy, marigold, Z. jujube, and Aloe vera) on the prevention and treatment of nipple pain and fissures. For assessing the effectiveness of Aloe vera, only one study was included in the review process and reported that *Aloe vera* is more effective than lanolin for treating nipple fissures [5]. The systematic search in this article has not been conducted accurately, many articles related to *Aloe vera* have not been included, and no meta-analysis has been performed. Therefore, a general conclusion cannot be reached by the results of one study.

Similar to a previous study, Asadi et al. conducted a systematic review to assess the effect of herbal medicine (eg., menthol, Aloe vera, cotton soaked, lanolin cream, peppermint cream, Ziziphus jujuba fruit, and saqez ointment) on the prevention and treatment of nipple trauma and pain of Iranian women. Authors searched the Iranian indexed publications in English and Persian until December 2017, and 11 articles were included in the systematic review. Among included articles, only 2 articles assessed the effect of *Aloe vera* on nipple pain, which shows a positive effect. Also, this article has not performed meta-analysis [28].

Also, in 2016, Hekmatpou et al. conducted a systematic review of 23 randomized control trials to evaluate the effectiveness of *Aloe vera* on the prevention of skin ulcers and treating burn wounds, psoriasis, postoperative wounds, genital herpes, chronic wounds, and cracked nipples. Their study's search period was from 1990 to 2016 with Persian and English language limitations. They were reported that *Aloe vera* has a positive effect on the prevention and healing of skin wounds and cracked nipple. In their study, only two articles include the review process regarding the effect of *Aloe vera* on the cracked nipple, and we are not doing a meta-analysis. On the other hand, the quality of the included study has not been assessed. Lack of assessing all resources and gray literature for systematic search has led to some relevant articles not being included in the systematic review [29].

Also, Pezeshki et al. in 2020 performed a systematic review of 6 interventional studies. Their study examined the effect of *Aloe vera* extract, breast milk, calendit-E, curcumin, lanolin, olive oil, and purslane on the healing of breast fissures in lactating women. Their results show that *Aloe vera* is more effective than olive oil and rubbing milk on the nipple fissures. But they don't perform any meta-analysis, and only 2 studies have assessed the effect of *Aloe vera* [30]. Moreover, in another systematic review that was conducted by Niazi et al. in 2021, the effects of topical treatment for the prevention and relief of nipple fissures and pain in lactating mothers were assessed. They have included 3 articles related to Aloe vera, and they show the positive effect of *Aloe vera* on nipple fissures, but they also did not perform a meta-analysis [31].

### 4.1. Strengths

1. Reviewing previous systematic review studies revealed that an accurate systematic search was not conducted in some studies, as well as none of them performed a meta-analysis and had only qualitative reports. This study is the first meta-analysis regarding the effect of *Aloe vera* on irritation and nipple pain in lactating women.
2. Searching in a large number of electronic databases, as well as hand-searching to yield maximum relevant articles in this field.
3. The homogeneity of studies that included the irritation outcome and their high quality.
4. High heterogeneity was seen among the included studies; therefore, a sensitivity analysis was performed to reduce the level of heterogeneity. After sensitive analysis, the level of heterogeneity in nipple pain was decreased.

Despite the study's strengths, the results have inherent limitations. The quasi-experimental studies were included in the systematic review. Furthermore, a meta-analysis of only seven articles was too small to prove our hypothesis. Therefore, these results need to be interpreted with caution.

## 5. Conclusion

Our result showed that *Aloe vera* can be considered a choice for relieving breast pain or irritation in lactating women compared with routine care or another treatment. Considering the limited number of studies conducted on this topic and the low sample size, future studies with a larger sample will be required to draw better conclusions. Future studies with large sample sizes will be required to draw better conclusions.

## Figures and Tables

**Table 1:** Characteristics of studies included in the systematic review.

Author	Year	Location	Study	Participants	Sample	Intervention	Tools	Outcomes	*P* value
Alamolhoda [14]	2019	Iran	RCT	Primiparous with breast soreness and irritation	*G*1= 55 *G*2= 55	Group 1= 0.5 ml *Aloe vera* gel/three times/Day Group2: breast milk	Pain= visual analog scale Irritation= store scale	Nipple pain, irritation, discharge follow up = 10, 14 days	*P* < 0.001

Meng [25]	2015	China	RCT	Patients with puerperal milk stasis	*G*1= 50 *G*2= 52 *G*3= 50	*G*1 = breast massage with cactus + *Aloe vera* cold compress *G*2= *Aloe vera* cold compress *G*3= traditional breast massage	Pain= visual analog scale	Pain and hardness follow up = after the treatments	*P* < 0.001

Eshgizade [26]	2016	Iran	RCT	Patients with nipple fissure	*G*1= 30 *G*2= 30 *G*3= 30	*G*1= 0.5 ml olive oil *G*2= 0.5 ml *Aloe vera* extract + 3–4 drop breast milk/3 times/day *G*3= 3–4 drop breast milk	Pain= visual analog scale Irritation= store scale	Pain and intensity of breast fissure follow up = 3, 7 days	*P* < 0.001

Sari [23]	2021	Indonesia	Quasi-experimental	Patient with breast engorgement	*G*1= 17 *G*2= 17	*G*1= *Aloe vera* cold compress *G*2= control	Pain= visual analog scale	Pain follow up = after the treatments	*P* < 0.01

Susanti [24]	2017	Indonesia	Quasi-experimental	Patient with breast engorgement	*G*1= 16 *G*= 16	*G*1= *Aloe vera* cold compress *G*2= control	Pain= visual analog scale	Pain follow up = after the treatments	*P* < 0.01

Tafazoli [4]	2009	Iran	RCT	Patient with breast soreness and irritation	*G*1= 50 *G*1= 50	*G*1= *Aloe vera* gel/three times/day *G*2= lanolin	Store scale = irritation = store scale	Irritation, follow up = 3, 7 days	*P* < 0.01

Saeidi [27]	2015	Iran	RCT	Patient with breast soreness and irritation	*G*1= 50 *G*1= 50	*G*1 = *Aloe vera* gel/three times/day *G*2= lanolin	Store scale = irritation = store scale	Irritation, follow up = 3, 7 days	*P* < 0.01

## Data Availability

The data used to support the findings of this study are available upon request.
